# Joint association of sleep duration and depression with new-onset hearing loss: a national cohort study

**DOI:** 10.3389/fnut.2025.1528567

**Published:** 2025-03-14

**Authors:** Fang Wang, Yu-Jun Xiong, Da-Ming Shao, Tian Lv, Shiqin Chen, Qian-Yuan Zhu

**Affiliations:** ^1^Department of Otolaryngology, Xinyang Central Hospital, Xinyang, Henan, China; ^2^Department of Gastroenterology, Beijing Hospital, National Center of Gerontology, Institute of Geriatric Medicine, Chinese Academy of Medical Sciences, Beijing, China; ^3^The University of Chicago Medical Center, Chicago, IL, United States; ^4^Department of Neurology, Zhuji Affiliated Hospital of Wenzhou Medical University, Zhuji, China; ^5^Department of Neurology, Yuhuan Second People’s Hospital, Yuhuan, China; ^6^Department of Neurology, Fenghua Hospital of Traditional Chinese Medicine, Ningbo, China

**Keywords:** CHARLS, hearing loss, depression, sleep, mediating effect

## Abstract

**Background:**

Hearing loss, a global health burden, is closely associated with depression and sleep disorders. However, the combined effects of sleep duration and depression on hearing loss risk remain unclear.

**Methods:**

Data from the China Health and Retirement Longitudinal Study (CHARLS) were analyzed, including 6,374 adults aged 45 and older. Cox proportional hazards models assessed the relationship between depression, sleep duration, and hearing loss. Mediation analysis explored the potential mediating roles of CESD-10 score and sleep duration on new-onset hearing loss. Subgroup analyses by age, sex, and BMI were also conducted.

**Results:**

Over a 7-year follow-up, 1,422 participants developed hearing loss. Both short sleep duration and high CESD-10 scores were independently associated with increased risk of hearing loss. Participants with long sleep duration but depression had a hazards ratio (HR) of 1.59 (95% CI: 1.35, 1.87) for hearing loss. Mediation analysis showed that sleep duration mediated 10.1% of the association between CESD-10 score and hearing loss, while CESD-10 score mediated 70.8% of the relationship between sleep duration and hearing loss.

**Conclusion:**

This study highlights the significant and interconnected roles of sleep duration and depression in the development of hearing loss. Interventions addressing both sleep and depression may offer more effective strategies for preventing and managing hearing loss.

## 1 Introduction

New-onset hearing loss is an emerging global public health concern, particularly prevalent among older adults (1). Epidemiological studies highlight hearing impairment as the third most common cause of years lived with disability, affecting 1.57 billion people worldwide (2). In China, the prevalence of hearing loss among middle-aged and older adults is approximately 45%, impacting nearly half of this population segment (3). Population aging and socioeconomic factors significantly influence the prevalence, severity, and overall disease burden of hearing loss (4), which may be attributed to the lifestyle of middle-aged and elderly populations in China, such as diet, environmental pollution, noise exposure, etc. This significant prevalence underscores the urgent need to address challenges related to aging and implement strategies to improve the quality of life for older adults. The high burden of hearing impairment presents not only medical challenges but also socioeconomic and public health implications, emphasizing the importance of targeted research and interventions to mitigate its impact.

Depression, a common mental health disorder, is linked to numerous chronic conditions and may adversely impact hearing health through both physiological and psychological pathways (5, 6). Cultural and social factors may contribute to a bidirectional relationship between hearing loss and depression. Social stigma and communication difficulties often lead to social withdrawal, loneliness, and psychological distress, which can exacerbate depressive symptoms (7). Depression, in turn, may increase the risk of hearing loss through physiological mechanisms such as chronic stress, inflammation, and vascular dysfunction, all of which can impair auditory function (8, 9). Limited access to healthcare and assistive devices, as well as workplace challenges and financial stress, may further worsen both conditions (10). However, research examining the association between depression and hearing loss is relatively scarce, and existing studies are predominantly from Western countries. There is a lack of comprehensive evidence focused on East Asian populations, particularly older adults in China.

Sleep, essential for maintaining physical and mental health, has also been implicated in auditory health. Poor sleep quality has been associated with various adverse outcomes, including impaired hearing (11). Mechanisms may include disrupted blood flow and reduced blood flow to the auditory system, potentially causing loss of outer hair cells (12). Nonetheless, studies investigating the relationship between sleep duration and hearing loss remain limited, and findings are inconsistent.

While depression and sleep disturbances frequently coexist and may share overlapping biological pathways, their combined effects on hearing loss have not been thoroughly examined. Additionally, it remains unclear whether sleep duration mediates the association between depression and hearing loss or whether depression mediates the impact of sleep duration on auditory function. Additionally, although previous studies have examined the associations between depression, sleep disturbances, and hearing loss, most of this research has been conducted in Western populations, with limited evidence from East Asian cohorts (13). Cultural, genetic, and healthcare system differences may influence the relationship between these factors, yet few studies have specifically analyzed how these associations manifest in Chinese adults (14). Given these gaps, the present study aims to: (1) investigate the independent associations of depression and sleep duration with new-onset hearing loss; (2) explore their joint effects on hearing loss risk; and (3) assess their potential mediating roles in each other’s relationship with hearing loss. Using nationally representative longitudinal data from the China Health and Retirement Longitudinal Study (CHARLS), our findings will provide a deeper understanding of the interaction between sleep, mental health, and auditory health, informing strategies for hearing loss prevention and management.

## 2 Materials and methods

### 2.1 Study design and participants

This study is a secondary analysis of the China Health and Retirement Longitudinal Study (CHARLS), a national, population-based cohort targeting Chinese adults aged 45 and above.^1^ The sample was drawn from 150 counties or districts and 450 villages across 28 provinces in China, spanning the period from 2011 to 2020 (15).

**Figure 1:**
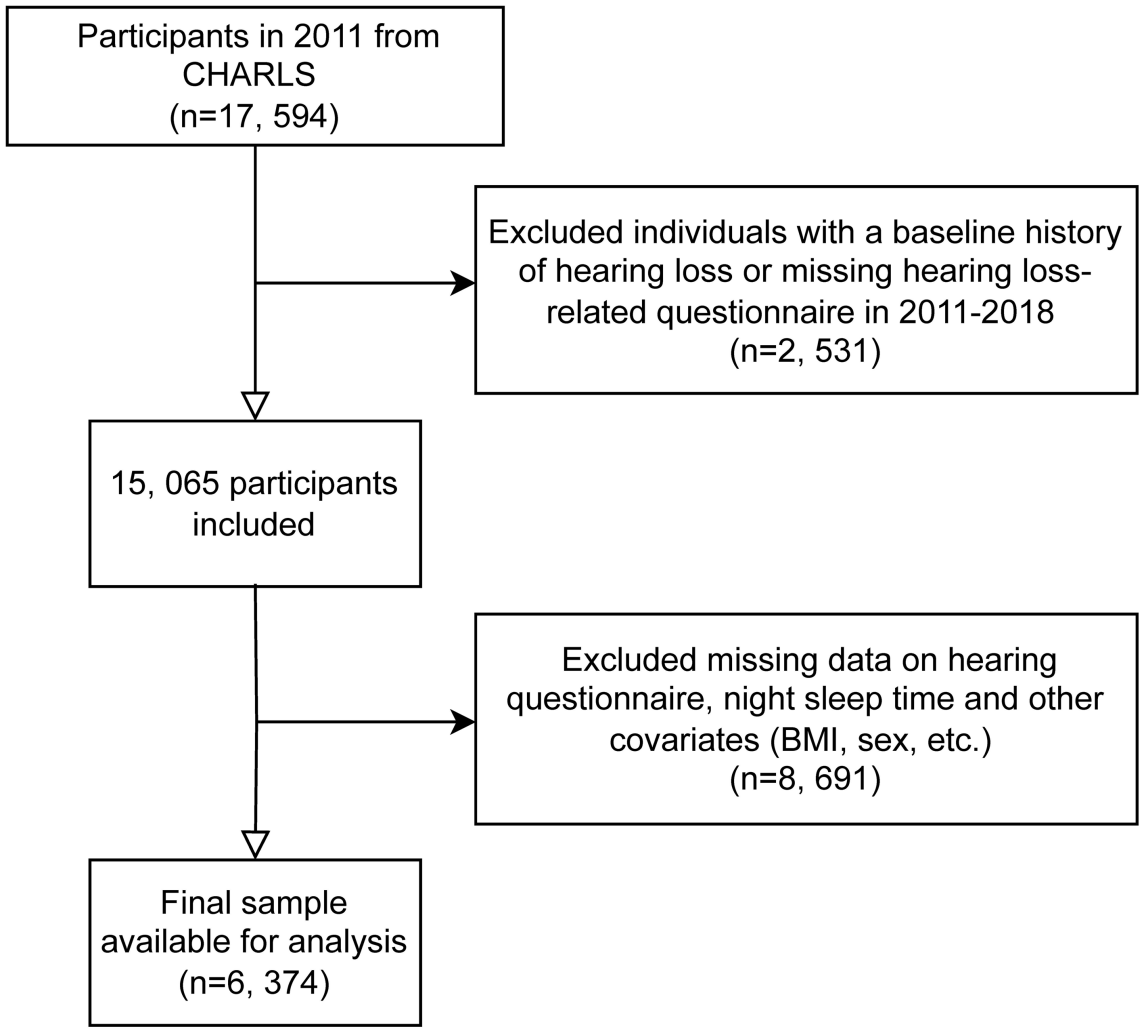
Flowchart of participant screening.

For our analysis, we utilized data from waves 1 to 4 of CHARLS, covering the period from 2011 to 2018. Data from wave 5 (conducted in 2020) was excluded due to the impact of COVID-19. Wave 1 in 2011 included 17,596 participants; individuals without baseline hearing data or those reporting poor hearing at baseline were excluded. Subsequently, during follow-up from 2013 to 2018, participants with missing data on hearing questionnaires, nighttime sleep duration, the 10-item Center for Epidemiologic Studies Depression Scale (CESD-10), or other covariates were also excluded. The exclusion criteria for this study included participants with missing data on key variables such as educational attainment, alcohol consumption status, hemoglobin levels, smoking status, diabetes mellitus diagnosis, residential status, uric acid levels, heart disease-related information, and other missing covariate data. These exclusions were necessary to ensure the integrity and completeness of the dataset, allowing for more accurate and reliable statistical analysis (Figure 1).

### 2.2 Assessment of depression and night sleep duration

Depressive symptoms were evaluated using the short version of the Center for Epidemiologic Studies Depression Scale (CES-D), a commonly employed self-reported tool for assessing depression in general populations (16). This scale comprises 10 items, each scored on a 4-point scale from 0 (rarely or not at all) to 3 (almost all the time). Participants with a total score of 10 or above were considered to have depressive symptoms (17, 18). Nighttime sleep duration was measured by asking participants: “During the past month, how many hours of actual sleep did you get at night (average hours for one night)?” Following prior studies, sleep duration was divided into two categories: short (<7 h) and long (≥7 h) (19).

### 2.3 Assessment of new-onset hearing loss and their follow-up time

Hearing status was evaluated through participant interviews, including questions such as: “Is your hearing very good, good, fair, poor, or very poor (with a hearing aid if you normally use it and without if you normally do not)? Would you say your hearing is excellent, very good, good, fair, or poor?” Hearing loss was defined when participants responded with “poor” (20, 21). This approach ensures a standardized assessment consistent with previously validated methods, allowing for a reliable determination of self-reported hearing loss (20). The onset of hearing loss was recorded as the time of the initial diagnosis.

The occurrence of hearing loss was calculated in different cases. For participants who did not report hearing loss at their most recent follow-up, the event timing was determined as the difference between the year of the last survey and the baseline year. For those who did develop hearing loss, the timing was based on the difference between the earliest reported year of hearing loss onset and the baseline year (22).

### 2.4 Covariate

According to prior research and clinical experts, potentially confounding and modifying variables were identified as follow: age, sex (male or female), residence (urban, rural), education (less than high school, high school, college). Clinical indicators such as uric acid, hemoglobin, blood lipids and glucose were measured in the laboratory. Heart disease, dyslipidemia and diabetes mellitus were evaluated through a standardized questionnaire that inquired whether participants had ever been diagnosed by a doctor with these conditions (23). Alcohol drinking status was classified into two distinct categories as ever/present or never. Smoke status was defined as former smoke but now quit, still smoke and never smoke (24, 25).

### 2.5 Statistical analysis

Data were presented as means and standard deviations (SDs) for continuous variables with normal distributions and as medians with interquartile ranges for those that were non-normally distributed. Categorical variables were described as frequencies with percentages. Baseline characteristics between groups were compared using the chi-squared test, ANOVA, or the Kruskal–Wallis rank-sum test, depending on the type of data (26).

We calculated the follow-up person-time for each participant, starting from the baseline survey (2011–2012) until either the date of hearing loss diagnosis or the end of follow-up (2017–2018), whichever occurred first. Cox proportional hazard regression models were used to estimate hazard ratios (HRs) and 95% confidence intervals (CIs) for outcomes associated with depression and sleep duration. Three models were developed: Unadjusted model as Model 0; Model 1 adjusted for age, sex, BMI, education, smoking, and alcohol consumption; Model 2 included the adjustments from Model 1 plus diabetes history, uric acid, dyslipidemia, hemoglobin, and residence. We also used 3-knot restricted cubic spline (RCS) regression to explore potential nonlinear associations.

**Figure 2:**
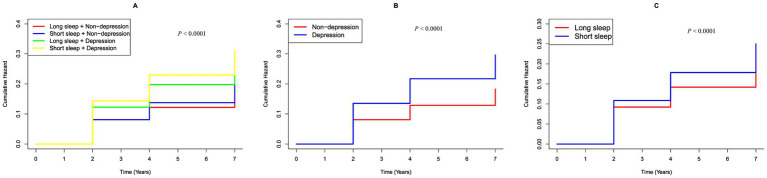
K–M plot of hearing loss by CESD-10 score and sleep duration subgroups. **(A)** Categorized by joint variable of CESD-10 score and sleep duration; **(B)** Categorized by CESD-10 score; **(C)** Categorized by sleep hours.

To evaluate the combined effects on hearing loss, participants were stratified into four groups based on their sleep duration (sleep hours <7 h and ≥ 7 h) and depressive status (categorized as non-depression and depression). In these groups, hazard ratios (HRs) for hearing loss incidence were calculated, using individuals with sleep hours ≥7 h and non-depression in quartile 1 as the reference group. We used the Kaplan–Meier survival curve to estimate the median hearing loss-free survival time of the population (Figure 2) and conducted a multivariable Cox regression analysis to examine associated risk factors (Table 1).

**Table 1:** Risk classification of hearing loss based on sleep duration and depression by multiple Cox regression analysis.

	Model 0	Model 1^a^	Model 2^b^
CESD-10 score	1.05 (1.04, 1.06)***	1.05 (1.04, 1.06)***	1.04 (1.04, 1.05)***
Normal	Ref	Ref	Ref
Depression	1.74 (1.57, 1.93)***	1.64 (1.47, 1.82)***	1.59 (1.43, 1.77)***
Sleep duration	0.92 (0.89, 0.94)***	0.95 (0.92, 0.97)***	0.95 (0.92, 0.97)**
Long	Ref	Ref	Ref
Short	1.30 (1.18, 1.45)***	1.18 (1.07, 1.31)**	1.19 (1.07, 1.32)**
Joint variable			
Q1	Ref	Ref	Ref
Q2	1.20 (1.04, 1.38)**	1.10 (0.95, 1.27)	1.11 (0.96, 1.28)
Q3	1.70 (1.45, 2.00)***	1.63 (1.38, 1.92)***	1.59 (1.35, 1.87)***
Q4	2.00 (1.74, 2.29)***	1.75 (1.53, 2.01)***	1.72 (1.50, 1.98)***

^a^Model 1 adjusted for age, sex, education, BMI, smoke, alcohol drink. ^b^Model 2 adjusted for age, sex, BMI, education, smoke, alcohol drink, diabetes mellitus, uric acid, dyslipidemia, hemoglobin, residence. **p < 0.01, ***p < 0.001. Q1: Sleep hours ≥ 7 h and non-depression. Q2: Sleep hours < 7 h and non-depression. Q3: Sleep hours ≥ 7 h and depression. Q4: Sleep hours < 7 h and depression.

A mediation analysis was conducted to evaluate the direct and indirect effects between depression and hearing loss through elevated sleep duration. The mediating role of depression on the relationship between sleep duration and hearing loss was similarly analyzed. To determine whether the associations between these factors and the risk of hearing loss varied by demographic characteristics, we assessed potential effect modification by age (<60 vs. ≥60 years), sex (women vs. men), BMI (≥28 vs. <28) and residence (urban vs. rural). All statistical analyses were performed using R software (version 4.2.1). Multiple imputations were conducted with the “charlsR” package. Mediation analysis utilized the “mediation” package, while Cox regression was carried out using the “survival” package. A two-sided *p*-value of <0.05 was considered statistically significant (27).

## 3 Results

### 3.1 Study participants and baseline characteristics

The final cohort consisted of 6,374 adults, of whom 1,422 were identified as having new-onset hearing loss (Table 2). The mean age was 57.76 ± 8.78 years, with males comprising 44.95% of the sample. In the group with new-onset hearing loss, a higher proportion of participants were older, had an elevated BMI, lower educational attainment, and a greater prevalence of heart disease. Additionally, more individuals resided in rural areas, had higher blood glucose levels, fewer reported alcohol consumption, shorter nighttime sleep duration, higher CESD-10 score, and a greater proportion of depressive symptoms compared to those without hearing loss.

**Table 2:** Baseline characteristics of participants.

	Overall (*n* = 6,374)	No hearing loss (*n* = 4,952)	Hearing loss (*n* = 1,422)	*p*-value
Age (years)	57.76 ± 8.78	56.82 ± 8.37	61.03 ± 9.37	<0.0001
Sex (male %)	2,865 (44.95)	2,218 (44.79)	647 (45.50)	0.66
BMI (kg/m^2^)	23.70 ± 3.85	23.83 ± 3.83	23.25 ± 3.88	<0.0001
Hemoglobin (g/dL)	14.36 ± 2.20	14.37 ± 2.21	14.32 ± 2.16	0.39
Education (%)				<0.0001
Less than high school	5,711(89.60)	4,369(88.23)	1,342(94.37)	
College	82 (1.29)	72 (1.45)	10 (0.70)	
High school	581 (9.12)	511(10.32)	70 (4.92)	
Residence				<0.0001
Rural	4,201(65.91)	3,163(63.87)	1,038(73.00)	
Urban	2,173(34.09)	1789(36.13)	384(27.00)	
Glucose (mg/dL)	109.10 ± 33.42	108.76 ± 33.29	110.30 ± 33.85	0.13
Uric acid (mg/dL)	4.42 ± 1.24	4.41 ± 1.23	4.44 ± 1.28	0.40
Dyslipidemia (yes %)	582 (9.13)	449 (9.07)	133 (9.35)	0.78
TC (mg/dL)	193.74 ± 38.37	193.40 ± 38.15	194.91 ± 39.09	0.20
HDL-C (mg/dL)	51.14 ± 15.13	51.01 ± 15.30	51.62 ± 14.51	0.17
LDL-C (mg/dL)	116.78 ± 35.04	116.46 ± 34.85	117.91 ± 35.69	0.17
TG (mg/dL)	131.80 ± 95.02	132.31 ± 96.34	130.00 ± 90.28	0.40
Sleep duration (hours)	6.41 ± 1.83	6.49 ± 1.78	6.15 ± 1.98	<0.0001
Smoke status (%)				0.30
Former, now quit	498 (7.81)	376 (7.59)	122 (8.58)	
Never	3,984 (62.50)	3,117 (62.94)	867 (60.97)	
Current	1892 (29.68)	1,459 (29.46)	433 (30.45)	
Alcohol drink (%)				<0.01
No	4,281 (67.16)	3,283 (66.30)	998 (70.18)	
Yes	2093 (32.84)	1,669 (33.70)	424 (29.82)	
Diabetes mellitus (%)	331 (5.19)	247 (4.99)	84 (5.91)	0.19
Heart disease = yes (%)	645 (10.12)	436 (8.80)	209 (14.70)	<0.0001
CESD-10 score	8.08 ± 6.16	7.54 ± 5.92	9.95 ± 6.63	<0.0001
Depression (%)	2,258 (35.43)	1,589 (32.09)	669 (47.05)	<0.0001
Follow up time (years)	6.32 ± 1.61	7.00 ± 0.00	3.95 ± 2.08	<0.0001

BMI, body mass index; TC, total cholesterol; HDL, high-density lipoprotein; LDL, low-density lipoprotein; TG, triglycerides; CESD-10, the 10-item Center for Epidemiologic Studies Depression Scale.

### 3.2 Correlation between depression, sleep duration, and new-onset hearing loss

**Figure 3:**
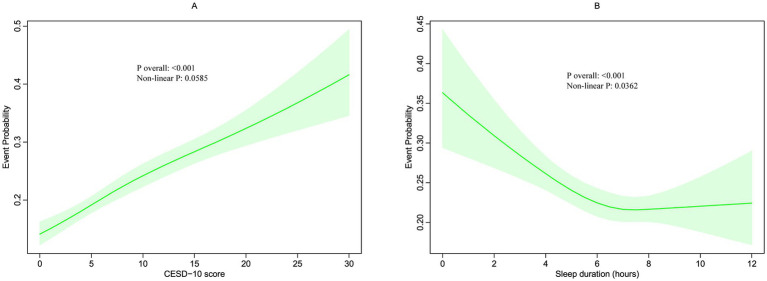
Restricted cubic spline (RCS) for the association between CESD-10 score **(A)** and sleep duration with the risks of hearing loss **(B)**.

The relationships between these factors and new-onset hearing loss were further examined using RCS curves, illustrated in Figures 3A,B. The RCS analysis demonstrated that the CESD-10 score, treated as a continuous variable, was significantly associated with an increased adjusted linear risk of hearing loss (*P* overall <0.001). Additionally, the relationship between sleep duration and hearing loss was significant but nonlinear (*P* overall <0.001, non-linear *p* = 0.0362), indicating that shorter sleep durations (<7 h) may be associated with a higher risk of hearing loss.

### 3.3 Associations of depression, sleep duration, and cumulative depression-sleep duration with new-onset hearing loss

During the follow up, 1,422 participants (22.31%) developed hearing loss. Figure 2 shows Kaplan–Meier curves illustrating the cumulative incidence of hearing loss among all participants. Participants with depression had a markedly higher risk of hearing loss compared to those without depression (*p* < 0.0001). Similarly, individuals with short sleep hours showed a significantly greater risk of hearing loss compared to those with long sleep hours (*p* < 0.0001). Furthermore, the combined presence of short sleep duration and depression was significantly associated with an increased risk of hearing loss when compared to participants with longer sleep durations and non-depression (*p* < 0.0001).

A multivariable cox regression analysis was then performed to assess the relationship between depression, sleep duration and their combined effect on hearing loss, as detailed in Table 1. The CESD-10 score, when analyzed as a continuous variable, was found to be associated with an elevated risk of hearing loss. Conversely, sleep duration showed a negative association with hearing loss, indicating that short sleep duration was linked to an increased risk of developing hearing loss. Initially, in the baseline model (Model 0), individuals in the depression group had a significantly higher risk of developing hearing loss compared to the normal group (*p* < 0.001). These associations remained significant after adjusting for age, sex, BMI, education, smoke, alcohol drink, diabetes mellitus, uric acid, dyslipidemia, hemoglobin, residence.

Similarly, when sleep duration was divided into two groups in the baseline model (Model 0), participants in the short sleep duration group exhibited a significantly higher risk of hearing loss compared to those in the long sleep duration group (*p* < 0.001). These significant associations persisted after adjusting for covariates in subsequent models (Model 1 and 2).

A joint analysis was performed to examine the combined impact of depression and sleep duration on the risk of hearing loss. The findings indicated that individuals with both short sleep duration and depression faced the highest risk of hearing loss (Table 1). Notably, participants with long sleep duration but with depression had a hazards ratio (HR) of 1.59 (95% CI: 1.35, 1.87) for hearing loss compared to those without depression and with long sleep duration, even after adjusting for covariates.

### 3.4 Mediation analyses of depression and sleep duration with hearing loss

**Figure 4:**
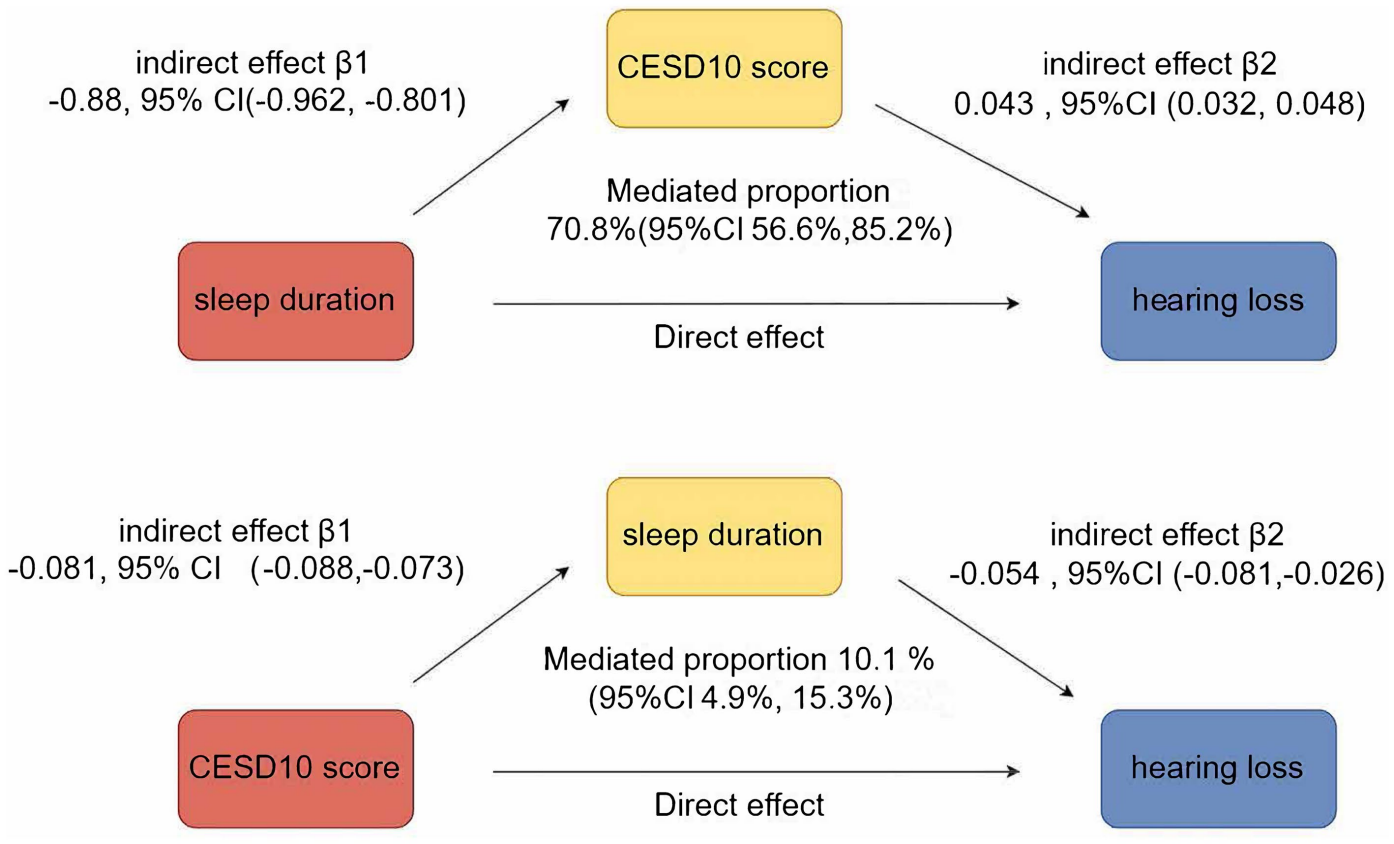
Mediation analyses of CESD-10 score and sleep duration on new-onset hearing loss.

Figure 4 illustrated the mutual mediation effects between sleep duration, depression, and hearing loss. Sleep duration significantly mediated 10.1% (95% CI 4.9–15.30%) of the association between CESD-10 score and hearing loss, while CESD-10 score simultaneously mediated 70.8% (95% CI 56.60–85.20%) of the association between sleep duration and hearing loss.

### 3.5 Subgroup analyses

The results of the subgroup analyses are presented in Table 3. Compared to participants with long sleep duration and no depression, those with short sleep duration and no depression exhibited a significant risk of hearing loss, particularly among individuals under the age of 60, with a BMI < 28, and who were male. Furthermore, participants with depression, regardless of sleep duration or characteristics such as age, BMI, residence, or sex, consistently showed a significantly higher risk of hearing loss.

**Table 3:** Subgroups analyses of the effect of sleep duration and depression on hearing loss.

	Q1	Q2	Q3	Q4	*P* for interaction
Age					0.267
≥60	Ref	1.046 (0.862, 1.269)	1.626 (1.306, 2.024)***	1.642 (1.369, 1.970)***	
<60	Ref	1.241 (1.002, 1.536)*	1.709 (1.346, 2.169)***	2.126 (1.735, 2.606)***	
BMI					0.658
≥28	Ref	1.273 (0.829, 1.955)	1.889 (1.179, 3.027)**	1.681 (1.088, 2.599)*	
<28	Ref	1.183 (1.016, 1.377)*	1.677 (1.413, 1.991)***	2.019 (1.750, 2.329)***	
Sex					0.074
Female	Ref	1.028 (0.837, 1.262)	1.504 (1.215, 1.862)***	1.750 (1.461, 2.097)***	
Male	Ref	1.383 (1.132, 1.690)**	2.032 (1.586, 2.603)***	2.413 (1.963, 2.965)***	
Residence					<0.001
Urban	Ref	1.165 (0.872, 1.557)	1.658 (1.114, 2.438)*	2.225 (1.641, 3.017)***	
Rural	Ref	1.139 (0.934, 1.387)	1.700 (1.365, 2.115)***	1.802 (1.490, 2.178)***	

*P < 0.05, **P < 0.01, ***P < 0.001. Q1: Sleep hours ≥ 7 h and non-depression. Q2: Sleep hours < 7 h and non-depression. Q3: Sleep hours ≥ 7 h and depression. Q4: Sleep hours < 7 h and depression.

## 4 Discussion

Our study suggests that both sleep duration and depression independently contribute to the risk of hearing loss, with potentially compounding effects when these factors co-occur. Previous studies investigating the link between sleep duration and hearing loss have reported inconsistent outcomes. For example, a study focusing on specific frequencies (1 kHz or 4 kHz) in Japanese adults reported a higher prevalence of hearing loss among those sleeping over 8 h per night, compared to those sleeping 6 h or <5 h per night (28). Another cross-sectional study in American adults suggested that both short and long sleep durations were linked to poorer hearing levels compared to sleeping 7–9 h (29). In contrast, a study involving thousands of Chinese adults in Zhejiang found that sleep duration over 8 h per night was inversely associated with hearing loss (30). What is more, in a Korean study, compared to participants sleeping ≤6 h, those with sleep durations of 7, 8, and > 8 h had a higher incidence of presbycusis by 24, 27, and 47% (12). However, the Dongfeng–Tongji Cohort Study identified an increased risk of hearing loss with sleep durations exceeding 9 h (31). Additionally, a U.S.-based study of 632 older adults found no significant link between sleep duration and hearing loss (32). Our findings provide valuable insight into the impact of sleep duration on hearing loss, offering additional evidence to this complex relationship.

Depression, another risk factor, has also been associated with auditory decline, likely due to its physiological impact on neuroendocrine pathways and inflammatory responses. A prior meta-analysis has revealed a bidirectional relationship between sensorineural hearing loss and depression (5). Hearing-impaired individuals often experience communication difficulties (33), social and emotional isolation (34), and mood disorders (35), all of which contribute independently to the onset of depressive symptoms. Furthermore, individuals with sensorineural hearing loss are frequently affected by varying levels of social isolation, which may further exacerbate depressive symptoms (36, 37). A population-based survey conducted in China also identified a positive correlation between self-reported hearing impairment and depressive status among older adults, in line with our findings. Moreover, participation in outdoor activities was suggested to mitigate the link between self-reported hearing loss and depression (38). Neuropsychological mechanisms underlying hearing loss have been associated with reductions in gray matter volume across 20 different brain regions (39), as well as atrophy of the auditory cortex (40) and reductions in both overall brain volume and right temporal lobe volume. Depression similarly induces smaller left hippocampal volume (41). This evidence strongly supports the potential benefits of interventions such as hearing aids and appropriate social engagement programs, which may significantly enhance the quality of life for older adults, improving both individual wellbeing and social outcomes (42).

Although research investigating the combined impact of sleep duration and depression on hearing health remains limited, preliminary studies suggest a synergistic effect. The reciprocal relationship between sleep disturbances and depression may exacerbate hearing loss risk beyond the independent contributions of each factor. Depression is known to disturb sleep patterns, resulting in fragmented and lower-quality sleep, which in turn can intensify depressive symptoms, creating a vicious cycle (43). This bidirectional relationship may amplify physiological impacts such as gut microbiota, serum metabolites, and inflammatory factors, leading to an increased vulnerability to sensory impairments. Importantly, this interaction underscores the need to consider both sleep and mental health in preventive and therapeutic strategies for hearing loss.

Our subgroup analyses revealed several notable findings. While both short sleep duration and depression were associated with an increased risk of hearing loss across all subgroups, the effects appeared to vary by BMI and gender. Interestingly, the association between short sleep duration and hearing loss was stronger among individuals with lower BMI (<28 kg/m^2^), while the risk associated with depression was more pronounced in males compared to females. Cultural and societal factors in China may have influenced our findings, particularly regarding rural–urban disparities. Access to healthcare services, including mental health support and sleep disorder treatment, differs significantly between urban and rural areas, potentially affecting both the prevalence and management of depression and sleep disturbances (44, 45). These findings suggest potential differences in physiological or behavioral responses to sleep disturbances and depression, warranting further investigation into underlying mechanisms such as hormonal regulation, metabolic factors, and sex-specific neurovascular pathways affecting auditory function.

Furthermore, our mediation analysis demonstrated that CESD-10 score mediated 70.8% of the association between sleep duration and hearing loss, indicating that depression plays a predominant role in linking sleep disturbances to auditory decline. This suggests that psychological distress and neuroinflammatory pathways may serve as key mechanisms through which inadequate sleep contributes to hearing impairment (46). The high proportion of mediation underscores the importance of addressing mental health alongside sleep interventions to mitigate hearing loss risk. Future studies should explore whether targeted psychological and behavioral interventions for depression, alongside sleep improvement strategies, can effectively reduce hearing impairment incidence in aging populations.

Given the significant associations observed between sleep duration, depression, and hearing loss, targeted interventions addressing these modifiable risk factors may help mitigate hearing impairment in middle-aged and older adults. First, improving sleep quality through behavioral and clinical interventions, such as cognitive behavioral therapy for insomnia (47), sleep hygiene education (48), and relaxation techniques (49), may help reduce the risk of hearing loss. Second, effective depression management, including psychotherapy, pharmacotherapy, and lifestyle modifications such as regular physical activity and social engagement, may provide additional benefits for auditory health (50). From the perspectives of public health polices, health education initiatives should be strengthened through community outreach programs, public awareness campaigns, and social media engagement to enhance knowledge about the interrelationship between sleep, mental health, and auditory function (51). Integrating sleep hygiene and mental health education into school curricula and workplace wellness programs can further reinforce these efforts. Concurrently, routine screening for sleep disturbances and depressive symptoms should be incorporated into primary healthcare services, utilizing community health workers and mobile health clinics to facilitate early identification and intervention, particularly in underserved populations. Since our findings suggest a potential mediating role of sleep duration and depression in hearing loss, a multidisciplinary approach integrating otolaryngology, neurology, psychiatry, and sleep medicine could optimize preventive strategies. Future clinical trials are warranted to assess the efficacy of such interventions in preserving hearing function and improving overall quality of life in aging populations.

Our findings carry substantial clinical implications. This study leverages data from the CHARLS, enabling a comprehensive investigation of the relationships between sleep duration, depression, and the onset of hearing loss in a large, nationally representative cohort of middle-aged and older adults in China. The CHARLS dataset provides valuable longitudinal data, allowing us to examine not only the independent effects of sleep and depression on hearing loss but also their combined influence. Our results indicate that participants with both short sleep duration and depressive symptoms exhibit the highest risk for new-onset hearing loss, even after adjusting for potential confounders such as age, BMI, and cardiovascular health factors. These findings highlight the potential importance of interventions targeting both sleep quality and mental health support in mitigating the risk of hearing loss in this demographic.

Our study has several limitations that should be acknowledged. First, the findings are specific to the Chinese population aged over 45 and may not be generalizable to other ethnic groups and younger age population. Second, although we controlled for a variety of potential confounders, the results could still be influenced by unmeasured factors, such as medication use, disease duration, or family history. Third, our study did not explicitly test for linearity assumptions in the relationships between sleep duration, depression, and hearing loss. Finally, the diagnosis of hearing loss, CESD-10 score, and sleep duration in surveys were primarily based on self-reported questionnaires rather than laboratory examinations, with no further objective assessments, such as audiometric testing, conducted to determine the specific degree of hearing impairment. This approach may carry a degree of subjectivity and recall bias, potentially compromising its accuracy due to reliance on individuals’ personal perceptions and interpretations. Future studies incorporating objective measures, such as audiometric tests for hearing loss, clinical diagnoses of depression, and actigraphy or polysomnography for sleep patterns, are necessary to validate and strengthen the findings.

Further research is warranted to explore the underlying mechanisms that link sleep, depression, and auditory health. Longitudinal studies focusing on biochemical markers of inflammation and vascular health, as well as neuroimaging studies examining structural changes in auditory pathways, may shed light on the complex interactions between these factors. Additionally, clinical trials assessing the effectiveness of interventions targeting both sleep and depressive symptoms may offer insights into comprehensive strategies to mitigate hearing loss risk. By addressing these modifiable risk factors, future work could pave the way for targeted and potentially more effective hearing loss prevention efforts in aging populations.

## 5 Conclusion

In conclusion, current evidence underscores the significant and interconnected roles of sleep duration and depression in the development of new-onset hearing loss. Shorter sleep duration has been associated with an increased risk of hearing impairment, while depressive symptoms have similarly shown a positive correlation with the onset of hearing loss. The bidirectional nature of the relationship between sleep patterns and depression highlights the necessity of incorporating both sleep management and psychological support in the prevention and treatment strategies for hearing loss. Further investigation into these underlying mechanisms, as well as prospective longitudinal studies, may offer new insights for developing comprehensive, targeted interventions for managing new-onset hearing loss.

## Data Availability

The datasets presented in this study can be found in online repositories. The names of the repository/repositories and accession number(s) can be found in the article/supplementary material.

## Footnotes

^1^ http://charls.pku.edu.cn/
